# Leveraging long-read sequencing technologies for pharmacogenomic testing: applications, analytical strategies, challenges, and future perspectives

**DOI:** 10.3389/fgene.2025.1435416

**Published:** 2025-04-30

**Authors:** Alireza Tafazoli, Mahboobeh Hemmati, Mahboobeh Rafigh, Maliheh Alimardani, Faeze Khaghani, Michał Korostyński, Jason H. Karnes

**Affiliations:** ^1^ Department of Pharmacology and Toxicology, University of Toronto, Toronto, ON, Canada; ^2^ Department of Medical Genetics and Molecular Medicine, School of Medicine, Mashhad University of Medical Sciences, Mashhad, Iran; ^3^ Medical Genetics Research Center, Faculty of Medicine, Mashhad University of Medical Sciences, Mashhad, Iran; ^4^ Student Research Committee, Faculty of Medicine, Mashhad University of Medical Sciences, Mashhad, Iran; ^5^ Department of Pharmaceutical Biotechnology, School of Pharmacy, Guilan University of Medical Sciences, Rasht, Iran; ^6^ Laboratory of Pharmacogenomics, Department of Molecular Neuropharmacology, Maj Institute of Pharmacology Polish Academy of Sciences, Kraków, Poland; ^7^ Department of Pharmacy Practice and Science, University of Arizona R. Ken Coit College of Pharmacy, Tucson, AZ, United States; ^8^ Department of Biomedical Informatics, Vanderbilt University Medical Center, Nashville, TN, United States

**Keywords:** long-read sequencing, clinical pharmacogenomics, applications and challenges, third-generation sequencing, rare pharmacovariants

## Abstract

Long-read sequencing (LRS) was introduced as the third generation of next-generation sequencing technologies with a high accuracy rate in genomic variant identification for some of its platforms. Due to the structural complexity of many pharmacogenes, the presence of rare variants, and the limitations of genotyping and short-read sequencing approaches in detecting pharmacovariants, LRS methods are likely to become increasingly utilized in the near future. In this review, we aim to provide a comprehensive discussion of current and future applications of long-read genotyping methods by introducing the opportunities and advantages as well as the challenges and disadvantages of state-of-the-art LRS platforms for the implementation of pharmacogenomic tests in clinical and research settings. New approaches to data processing, as well as the challenges and pitfalls of performing such tests in daily practice, will be explored in detail. We provide references to resources for those who are interested or intend to employ LRS in pharmacogenomics screening, both in clinical and research settings.

## Introduction

The field of pharmacogenomics (PGx) is poised to integrate clinical genetic testing to predict the drug response into daily clinical practice (Borden et al., 2021). PGx testing is now available for many gene–drug interactions, which are now cataloged and curated in well-established databases (Barbarino et al., 2018; Cannon et al., 2024). PGx tests have the potential to enable drug-response stratification and implementation of personalized medicine across diverse populations. Many clinical and research centers are now considering PGx profiling in addition to existing genome screening and considering extraction of PGx markers out of exome or genome sequencing data (van der Lee et al., 2020).

Increased accuracy of genotyping approaches may add significant insights into treatment outcomes and prevent potential adverse drug-reactions (ADRs) (Golubenko et al., 2023). The PGx data interpretation workflow mainly relies on haplotype phasing, complex genomic region analysis for pharmacogenes, diplotype detection, allele imputation, and phenotype prediction. Although current genotyping methods mostly use array-based or short-read sequencing (SRS) technologies for single-nucleotide variant (SNV) detection within drug-related genes, more accurate approach(es) should be considered, especially for those genes with clear guidelines and actionable clinical recommendations.

**FIGURE 1:**
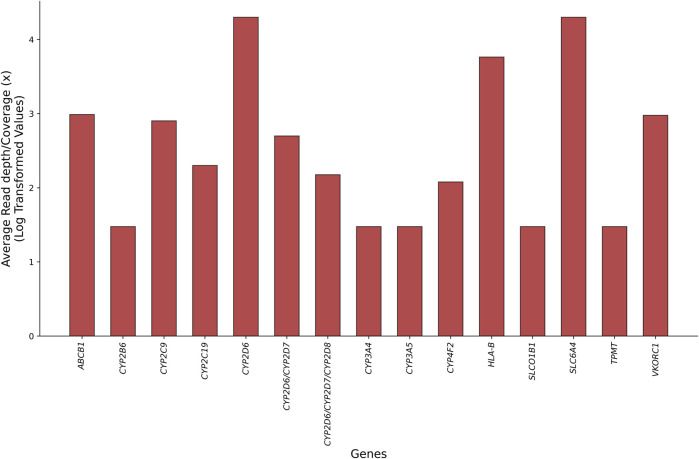
Comparison of average read-length distribution and coverage for core pharmacogenes sequenced by currently available long-read technologies within clinical pharmacogenomic tests to recall the ambiguous and novel genotypes (see the main text and Table 3 for references).

Pharmacogenes contain highly polymorphic and/or homologous regions, repeated variants in the non-coding part of the genome, or rare structural variants (SVs), such as large insertions or deletions and segmental duplications or homopolymers (van der Lee et al., 2022). The advent of long-read sequencing (LRS) technologies, known as third-generation sequencing approaches, and their integration into PGx investigations highlight opportunities for resolving such variations (Table1). From providing unambiguous alignment of genomic regions to addressing challenges in achieving uniform coverage of GC-rich positions, LRS platforms can perform accurate genotyping in analytically challenging pharmacogenes without specific needs for DNA treatment, and they can perform full phasing and resolve complex diplotypes while decreasing the occurrence of false-negative results in a single assay (Caspar et al., 2018; Turner et al., 2023). Figure 1 displays the read length and coverage for some core pharmacogenes, decoded by LRS technologies in clinical PGx setting.

**TABLE 1:** Pharmacogenes and their particular characteristics that would take advantage of long-read sequencing.

Genes	Challenging Features	References
*CYP2B6*	− Structural variants such as CYP2B6*29 and CYP2B6*30 − Pseudogenes such as *CYP2B7* − Repetitive sequences such as SINEs^1^	Desta et al. (2021), Turpeinen and Zanger (2012), Martis et al. (2013)
*CYP2D6*	− Structural variants such as CYP2D6*5 − Copy number variations such as CYP2D6*1xN, *2xN, and*4xN − Pseudogenes such as *CYP2D7* and *CYP2D8* − Repetitive regions such as REP6	Nofziger et al. (2020), Wang et al. (2024), Taylor et al. (2020)
*CYP2A6*	− Structural variants such as CYP2A6*4, and CYP2A6*2 − Pseudogenes such as *CYP2A7* − Repetitive regions such as SINEs and LINEs^2^	Martis et al. (2013), Langlois et al. (2024)
*CYP2E1*	− Structural variants, particularly duplications − Pseudogenes such as *SPRNP1* − Repetitive regions such as VNTRs^3^ − Repetitive regions such as SINEs	Martis et al. (2013), Tremmel et al. (2023), Chamorro et al. (2017)
*CYP4F2*	− Copy number variations and structural variants such as CYP4F2*16	Zubiaur et al. (2024)
*G6PD*	− Structural variants − Copy number variations	Sherman et al. (2024), Zhang et al. (2023)
*GSTM1*	− Structural variants − Copy number variations − Repetitive regions on both sides of the gene − Gene deletion polymorphisms (homozygous deletions)	Tremmel et al. (2023), Tremmel et al. (2020), Filippova et al. (2014), Bolt and Thier (2006)
*GSTP1*	− Rare copy number variations	Ramírez et al. (2019)
*HLA*	− Structural variants − Repetitive regions	Chen et al. (2008), Castelli et al. (2018)
*SLC22A2*	− Structural variants such as SLC22A2*S1 and SLC22A2*S2 deletions	Sherman et al. (2024)
*SULT1A1*	− Highly polymorphic copy number variations − Copy number variations − Repetitive sequences	Tremmel et al. (2023), Sherman et al. (2024), Wankaew et al. (2022)
*UGT1A4*	− Structural variants such as UGT1A4*S1 partial deletion	Sherman et al. (2024)
*UGT2B15*	− Structural variants such as partial gene deletion UGT2B15*S1 − Rare copy number variations − High sequence identity *UGT2B1* − Pseudogenes	Sherman et al. (2024), Tremmel et al. (2020), Romero-Lorca et al. (2015), Ménard et al. (2009)
*UGT2B17*	− Structural variants − Copy number variations such as *UGT2B17* gene deletion − High sequence identity with the *UGT2B* family of genes − Pseudogenes	Tremmel et al. (2023), Sherman et al. (2024), Tremmel et al. (2020), Romero-Lorca et al. (2015), Ménard et al. (2009)

The aim of this review is to introduce and highlight the current and potential applications of LRS in PGx profiling of individuals through discussion of its advantages alongside challenges and limitations. While other literature works addressed LRS for PGx testing in a general view (Neu et al., 2023), this review explores applications of LRS in PGx in greater depth and provides more topic-specific contexts. We start with a brief overview on technologies and up-to-date workflows for data processing and pharmacovariant identification within the LRS output. Then, we take a broader look at the opportunities of applying LRS in PGx studies. Several real-world examples of using LRS for decoding complex core drug-related genes, such as *CYP2D6 and HLA-B*, are illustrated in detail as a proof of principle. Finally, potential challenges and existing limitations for utilization of the technologies are explored and discussed. We believe the article constitutes a valuable reference for those who are interested or intend to employ LRS in PGx studies, both in clinical and research settings.

## Methods

A literature review was conducted to identify studies exploring the use of LRS technologies in PGx testing and clinical applications for articles published between 2010 and 2025. The search began with general studies on LRS technologies and their functionality, followed by a focused investigation using targeted search terms relevant to our review objectives and workflow. Comprehensive literature search: databases such as PubMed, Scopus, and Web of Science were searched, complemented by manual examination of reference lists from the selected publications. Search terms and combinations: [(“Long-read sequencing” OR “PacBio sequencing” OR “Nanopore sequencing” [Mesh]) AND “Pharmacogenomics” (Mesh)] AND (“Genetic Variation” [Mesh] OR “Drug Response Biomarkers, Genetics” [Mesh] OR “Drug Targeting” [Mesh] OR “Personalized Medicine” [Mesh]) [(“Long-read sequencing” OR “PacBio sequencing” OR “Nanopore sequencing”) AND (“CYP2B6” OR “CYP2C19”OR “CYP2C9” OR “CYP2D6” OR “CYP3A4” OR “CYP3A5” OR “DPYD” OR “SLCO1B1” OR “TPMT” OR “UGT1A1” OR “HLA” OR “VKORC1” OR “NAT1” OR “NUDT11”)]. For general terms, it was [(“Long-read sequencing” OR “PacBio sequencing” OR “Nanopore sequencing”) AND (“Genetic variation” OR “Polymorphism, genetic” OR “Genetic variation”) AND (“Gene identification” OR “Gene discovery”)]. Inclusion criteria included the following: a) studies investigating the application of LRS technologies (e.g., PacBio and Nanopore) in PGx; b) studies using LRS to analyze genetic variants associated with actionable genes, including drug response and drug target genes; c) studies evaluating clinical applications of LRS in PGx; d) studies decoding genetic variants in patients with adverse drug reaction-like clinical manifestations; e) publications in English with the full text available. Exclusion criteria included the following: a) studies not utilizing LRS for analyzing PGx-related genetic variants; b) studies focused on non-pharmacogenomic applications or unrelated diseases; c) publications not available in English or full text (e.g., conference abstracts); d) studies published before 2010, given the relatively recent development of the technology. Outcome measures included the following: a) studies demonstrating the application of LRS for decoding variants in actionable PGx genes and b) research using LRS to identify genetic variants in complex genomic regions, challenging to resolve with SRS or other standard assays. A total of 152 published articles met the inclusion criteria and were included in the current literature review.

### Long-read sequencing (a brief overview of technology and data processing)

#### Third-generation next-generation sequencing platforms

With continuous development of sequencing technologies and decrease in LRS costs, applications will likely shift toward third-generation sequencing platforms due to their longer read length and the capability for using real-time single-molecule sequencing approaches. The ability to read long stretches of DNA without the need for amplification minimizes errors introduced during the amplification process (Athanasopoulou et al., 2021). Recent versions of LRS technologies have also improved error correction algorithms that reduce the inaccuracy. As a result, LRS has the potential to become an essential tool for various applications, including genome assembly, structural variant detection, epigenetic analysis, and PGx profiling (Amarasinghe et al., 2020a). Available platforms now directly produce uninterrupted sequences of native DNA, ranging from 10 kilobases to megabases in length with an accuracy up to 99.9% (Sequel II and PromethION) (Mantere et al., 2019; Pollard et al., 2018). In this review, we focus on two LRS technologies, Pacific Biosciences’ (PacBio) and Oxford Nanopore Technologies (ONT), which have been used extensively in genomic and PGx research. Table 2 provides an overview of these technologies, including the advantages and disadvantages specific to PGx profiling.

**TABLE 2:** Comparison of two commonly used long-read sequencing genotyping methods for PGx testing.

Technology	Instruments		Price	Read Length	Processing time	Prospects in PGx studies	Limitations	References
Pacific Biosciences (PacBio)-Single Molecule Real-Time (SMRT) Sequencing	Single-molecule real-time (SMRT)		∼$1,500 to $3,000 per sample	Between 10 and 50 kilobases	up to 30 h	Long read, full coverage of complex pharmacogenes, enhances genetic variation accuracy for drug response, phasing haplotypes (uncovering genetic variant interactions in drug response), and Revio system is proficient in detecting DNA methylation without bisulfite treatment	Expensive, varying accuracy (high error rate), lengthy turnaround time	Petersen et al. (2019), Satam et al. (2023), Salk Institute for Biological Studies., 2024 Pacific Biosciences., Revio System Brochure., 2023
	Sequel IIe		∼$1,500 to $3,000 per sample					
	HiFi (High-Fidelity)	Vega benchtop system	∼$1,500 to $3,000 per sample					
		Revio system						
Oxford Nanopore (Nanopore Sequencing)	Flongle		∼$4.50 per sample	Between 5 to 300 Kb, longer reads = up to 2.3 Mb, ultra-long = >4 Mb	up to 72 h	Long read (detection of structural variations, gene fusions, and complex genomic rearrangements, cost-effective, rapid TAT (turnaround time), full coverage of complex pharmacogenes	Less accuracy (high error rate)	Petersen et al. (2019), Blanco et al. (2020), Oxford Nanopore Technologies., 2024
	MinION		∼$10–$50 per sample					
	GridION $100-$275 per samplePromethION		∼$100–$1,000 per sample					

PacBio and ONT are LRS platforms that offer reads ranging from 50 kilobase pairs (kbp) to the current record of 2.3 million base pairs (Mb), respectively (Shendure and Ji, 2008). The accuracy of base-calling in both of these technologies has significantly improved in recent years, and it is now claimed that the raw base-called error rate has been lowered to <1% for PacBio and <5% for nanopore sequences (Payne et al., 2019). These error rates are computed by comparing the base calls generated by the sequencing platform to a known reference or consensus sequence. Various factors contribute to sequencing errors, including machine errors, chemistry limitations, and other technical challenges. Improvements in base-calling algorithms, sequencing chemistry, and overall technology advancements have collectively contributed to the reduction in these error rates over time (Amarasinghe et al., 2020a; Wenger et al., 2019).

The PacBio single-molecule real-time (SMRT) sequencing technology uses a circular DNA molecule template, referred to as SMRTbell, which has a unique topological structure. The SMRTbell is composed of a double-stranded DNA insert and single-stranded hairpin adapters located on both ends, facilitating effective and high-precision sequencing of DNA. The length of the DNA insert can range from 1 to 100 kilobases, making it possible to generate lengthy sequencing reads. After the successful assembly of the SMRTbell, a DNA polymerase binds to it before loading onto an SMRT Cell. The SMRT Cell, which houses up to 8 million zero-mode waveguides (*see the mini glossary*), is then utilized for sequencing (Kronenberg et al., 2018; Audano et al., 2019; Huddleston et al., 2017; Chaisson et al., 2015). Recently, PacBio developed two new platforms, named Revio and VEGA system, which promise highly advanced genome profiling approaches, such as direct detection of genome methylation in a very competitive turnaround time and/or a highly accurate LRS platform. ONT, on the other hand, enables the efficient and affordable analysis of extremely small amounts of DNA without manipulating cells (Ozsolak, 2012). While SMRT sequencers (RSII, Sequel, and Sequel II) recognize fluorescent signals, core nanopore sequencers (MinION, GridION, and PromethION) assess the variations in ionic current that arise as single-stranded nucleic acids traverse nanopores (Merker et al., 2018). A nanopore is a very small pore that is typically made of a solid material or protein embedded within a membrane and immersed in an electrolyte solution. The nucleic acid to be sequenced is passed through the nanopore, causing disruptions in the electrical current passing through the pores, and each base has a distinct effect on the current flow. These changes are recorded and analyzed. One advantage of nanopores is their portability for some sequencing devices (Stahl-Rommel et al., 2021; Burton et al., 2020; Castro-Wallace et al., 2017). Furthermore, DNA or RNA can be directly sequenced, whereas previous technologies required the synthesis of nucleic acids (Ozsolak, 2012; Marx, 2023). Recent efforts in ONT development have shifted their focus toward solid nanopores, with particular emphasis on materials like graphene and silicon. This shift is motivated by several factors, including the ability to achieve more precise control over the pore structure (Hall et al., 2010).

Both PacBio and ONT technologies are able to negotiate the most repetitive parts of the human genome. However, differences in their chemistry and sequence detection methods impact the length of their reads, the accuracy of the base pairs, and the amount of data they can produce (Kronenberg et al., 2018). Furthermore, the analysis of third-generation sequencing data necessitates use of specialized tools compared to second-generation platforms.

#### Bioinformatics of LRS technologies for PGx studies

##### Brief overview of PacBio and ONT data processing

The data generated by LRS platforms present unique challenges and opportunities for analysis. Routine workflow for LRS data analysis benefits from both common and LRS-specific tools, which may result in individual variant call format (VCF) or separate VCF files for different types of variants. On the other hand, mapping of long and sometimes noisy reads demands an accurate algorithm(s) of the seed-and-chain framework (*see the mini glossary at the end of this article*) for dealing with the initial 10%–20% error rates in some LRS reads. A valuable database is available to introduce all currently existing software designed for processing and analyzing LRS data, which is accessible through the web address https://long-read-tools.org/ (Amarasinghe et al., 2020a).

SMRT cells can be merged prior to assembly to achieve more coverage. PacBio comes with a basecall format referred to as bas.h5/bax.h5 (now POD5) or BAM file format as the primary output from SMRT instruments. To convert h5 or POD5 to Fastq files, several tools with different features have been developed, which may be used for multiple manipulations of PacBio data from basecalling to read alignment (Li, 2018). Other tools also have also been introduced recently and are available, outperforming in terms of sequence identity, the count of mapped bases, and runtime efficiency (Chaisson and Tesler, 2012; Hufnagel et al., 2020).

Previous ONT basecalling utilized an “official” state-of-the-art tool named *Guppy* for converting Fast5 format to Fastq. The tool was a neural network basecaller, which could also filter and remove low-quality reads, clip ONT adapters, and estimate the probability of methylation signatures for each base (Wick et al., 2019). However, recent advancements in nanopore sequencing include the transition to POD5 as the raw data storage format and *Dorado* as the default basecaller. These updates enhance data processing efficiency and accuracy. *Dorado* is a data processing toolkit that includes alignment, modified base detection, and barcode demultiplexing and normal basecalling. For modified bases, currently, it can identify 5mC, 5hmC, 4mC + 5mC, and 6mA for DNA and m6A and pseudouridine for RNA (https://nanoporetech.com/document/data-analysis; De Coster et al., 2018).

In addition to *Dorado*, which uses *Minimap2* as a built-in algorithm, genome alignment also applies general standalone tools (Sedlazeck et al., 2018a). However, such tools have high computational needs.

##### Phasing, haplotype phasing, and haplotype/diplotype imputation

Phasing in PGx testing refers to the process of assigning individual alleles in a diploid individual to specific copies of a chromosome. It is an essential part of PGx data processing because it identifies whether genetic variants associated with the drug response are located on the same or different alleles (*cis* or *trans*). This is often crucial for understanding compound heterozygosity and predicting an individual’s drug response and potential adverse reactions accurately. Correct phasing ensures personalized and effective medication strategies, maximizing the benefits of PGx testing. Haplotype phasing is a crucial step in the identification of diplotype determination and subsequent phenotype prediction for a PGx test. It can be the decisive factor in distinguishing between a poor metabolizer (characterized by two loss-of-function variants located on distinct alleles) and an intermediate metabolizer (defined by having two loss-of-function variants on a single allele with no variants on the opposite allele). Hence, improved accuracy of haplotype phasing in PGx has the potential to improve phenotype determination (van der Lee et al., 2020). Currently, *WhatsHap* and *HapCUT2* (by using sorted BAM files from *Minimap2*) are available for direct haplotype phasing of long-read WGS genotyping results. Genotype imputation and admixture analysis are also likely to benefit from improved phasing (Martin et al., 2016). The recently developed tool *LRphase* utilizes haplotype-resolved heterozygous variants, derived from various genomes including maternal and paternal genomes, in two different modes of scoring and matching to the observed genotype (Holmes et al., 2023).

##### PGx variant calling with LRS

We start this section with a summary of the tools applied for LRS variant calling as many of PGx variant callers accept the LRS VCF files as their input data for subsequent diplotype/phenotype prediction. The quality of the input data will have a significant impact on the accuracy for variant callers. Both PacBio and ONT require a minimum amount of coverage for read-based and/or assembly-based variant calling. However, the assembly-based method is preferred since the error correction step would be run during the genome assembly, thus reducing the false detection of SVs and making it a more appropriate choice for individualized sequencing of large and complex genomic regions (Lee et al., 2023).

SNVs and short insertion–deletions (InDels) in the LRS result can be identified by common callers or PacBio/ONT-related tools. Among the variant callers that were tested for LRS data from the Genome in a Bottle (GIAB) and HGSVC Freeze 4 resources (Helal et al., 2022; Byrska-Bishop et al., 2022), *DeepVariant* and *Clair3* have been evaluated for PGx variant calling and consistently demonstrated superior performance and robustness (van der Lee et al., 2022; Barbitoff et al., 2022). For SV detection, *Sniffles2* utilizes an innovative scoring system, which considers factors such as the position, size, type, and coverage of candidate SVs to exclude false calls. This approach effectively mitigates the high indel error rates in LRS results, ensuring precise detection of SVs in both germline and somatic variations in population-level analyses for PacBio and ONT read data (Sedlazeck et al., 2018a). Another common variant caller for LRS data is *SVIM* (pronounced swim), with the ability to mark and classify six types of SVs, namely, insertions, deletions, tandem duplications, interspersed duplications, inversions, and translocations. In addition, both PacBio and ONT platforms possess their own structural variant caller tools, identifying SVs within the related sequencing data. For instance, *SMRTlink pbsv,* which accepts pre-processed data named SvSIG, works with PacBio SMRT reads, while other tools such as *Evince* work with ONT reads (Bolognini and Magi, 2021).

Currently, ONT variant calling relies on NextFlow’s variant caller tool *human variation workflow* (*epi2me-labs/wf-human-variation*). It can perform basecalling of Fast5 and calling all types of variants in ONT reads, including SNVs, SVs, methylation signatures, CNVs, and short tandem repeats (STRs), simultaneously. The recently introduced tool *NanoCaller* uses deep learning and neural networks to call novel variants within complex genomic regions (Ahsan et al., 2021).

SV annotation databases like *AnnotSV* (https://www.lbgi.fr/AnnotSV/) may be utilized for the interpretation of potential pathogen SVs in human genome reads. The webserver identifies possible false-positive variants among all detected SVs and visualizes the range of variants present. It provides improved annotation from various sources, three output formats, innovative user interfaces, including an interactive Circos view, and SV scoring (0–1) based on human phenotype ontology (HPO) and Exomiser (Geoffroy et al., 2023). However, once the phased LRS-VCF files are ready, PGx-dedicated bioinformatic tools and algorithms with the capacity to accept LRS reads as the input data must be applied for accurate diplotype determination and subsequent phenotype prediction. The data may also be used to predict a PGx phenotype through the assignment of a “gene activity score.” This score is assessed by calculating the sum of individual activity scores assigned to each allele, and when there is more than one copy of an allele, the score is multiplied by the number of copies (Charnaud et al., 2022).

Numerous PGx tools are available for star allele calling using sequence data. Recently developed tools demonstrate promise in star allele calling and phenotype estimation in NGS data, including WGS (Tafazoli et al., 2022). Here, we provide a brief overview of the tools with the capability of processing LRS-VCFs for allele imputation and diplotype assignment in clinically important pharmacogenes. *Aldy* (version 4.0.1 and more) announced the adaptation with the LRS result in its latest update. While other tools such as *Stargazer*, *Astrolabe*, *PharmCAT*, *StellarPGx*, and *Pnno* do not support LRS reads natively, *Aldy4* accepts different types of sequencing data, including LRS, using new phasing strategies and improved variant calling models. Tested by two PacBio HiFi datasets and compared with other tools, *Aldy4* was found to demonstrate superior performance than other tools in the detection of the star alleles, except for a small number of *CYP2D6* fusion and CNV alleles and one *CYP2C9* call, where discrepancies were observed. However, finding and phasing novel unreported alleles within pharmacogenes like *CYP2C19*, *CYP2B6*, *CYP3A4*, *DPYD*, and *SLCO1B1* is considered a major advantage for *Aldy4* when applying for LRS variant calling (Hari et al., 2023).

Another computational algorithm with built-in compatibility for decoding LRS variants is *Cyrius*. The tool demonstrated its unique ability to call both SNVs and known SVs in *CYP2D6,* including *5, *13, *36, *68, and duplications. However, currently, *Cyrius* is only adapted for star allele calling within *CYP2D6* and its pseudogene *CYP2D7* (Chen et al., 2021). Other bioinformatic solutions such as *Stargazer* and *PharmCAT* may be used for LRS despite limitations for the identification of some distinct SV alleles (Lee et al., 2019). The standard pipeline must be provided phased input data, using BAM/CRAM (e.g., to create GDF for *Stargazer*), GVCF, and VCF files. The workflow itself may be modified by adding additional parameters (similar to “-S” in *PharmCAT*) when there are pooled multi-coverage samples. Merged VCF files also require separation in batches by tools like *BCFtools*. The *Stargazer* tool has now been superseded by PyPGx (Lee et al., 2022). This Python package works with both SRS and LRS alongside SNVs from SNP array data. Through a machine learning-based approach, PyPGx will predict star-allele diplotypes and phenotypes. This promised completely free and open-source package supports both the command line interface (CLI) and application programming interface (API) along with comprehensive and supportive documentations. Compatible with both the common genome builds (hg19 and hg38), PyPGx covers 87 pharmacogenes. The tool is highly appreciated and used by many reference centers and projects, as well as the genetic testing reference materials coordination program (GeT-RM), a program under the Centers for Disease Control and Prevention (CDC), and the 1000 Genomes Project (1KGP) (N = 2,504) (PyPGx documentation, accessed on 03-04-2025). Supplementary Table S1 provides details of currently available tools for converting raw LRS data into related VCFs and PGx annotated output.

### Applications and opportunities

**FIGURE 2:**
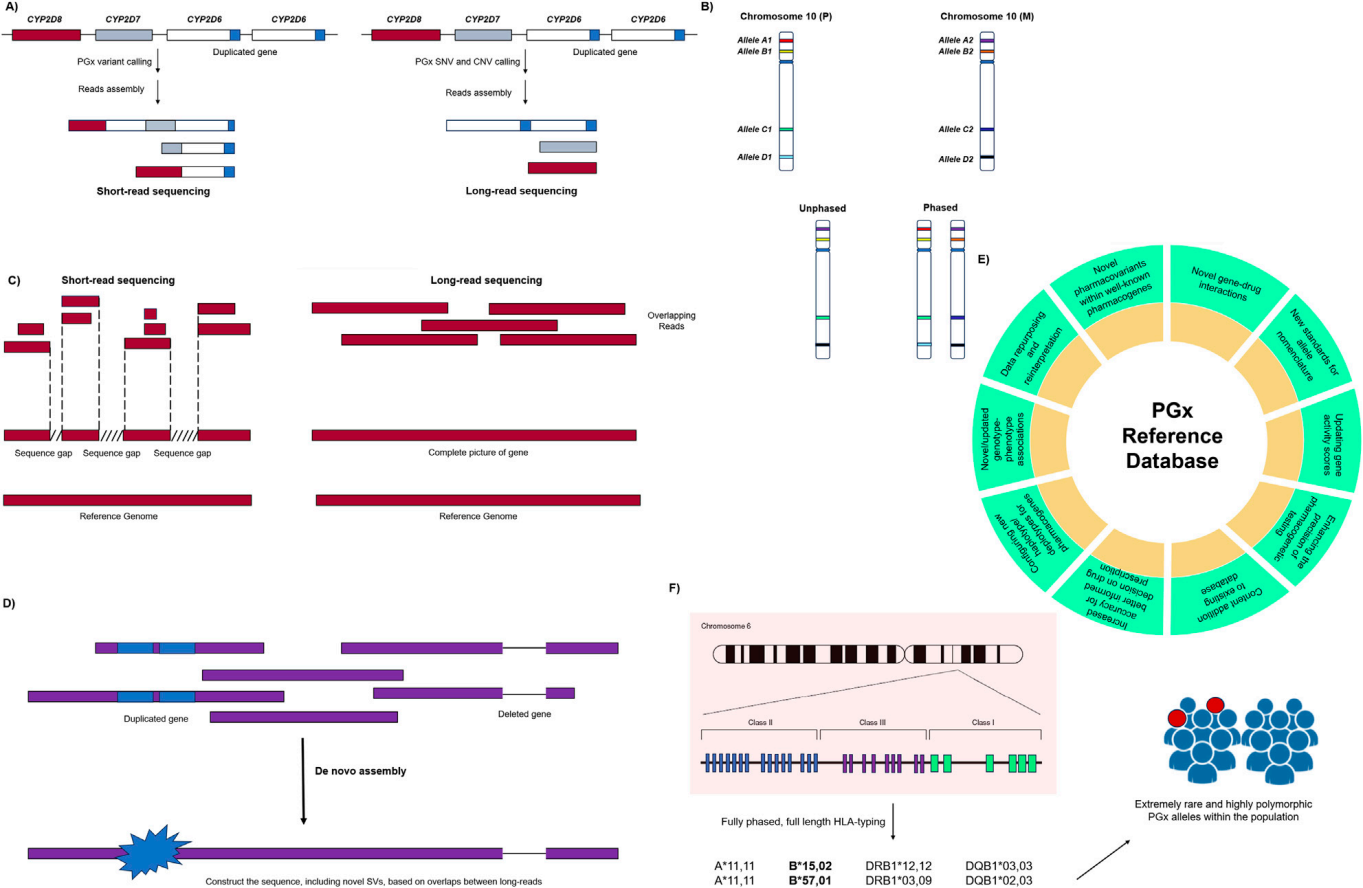
Main applications of long-read sequencing technologies in pharmacogenomic testing. **(A)** Decoding complex pharmacogenes: LRS may provide more accurate sequencing reads compared to the short-read sequencing result for the genomic region with high polymorphic and structural rearrangement. This leads to correct variant calls while dealing with CNVs and other SVs in core pharmacogenes. **(B)** Haplotype characterization and phasing: LRS makes the true allele decomposition, which may lead to the comprehensive picture of genome for target pharmacogenes. This includes assigning the right position of each allele on paternal/maternal chromosomes for better understanding of allele inheritance and haplotype definition. **(C)** Resolving ambiguous genotype calls: short-read sequencing may result in some ambiguity while trying to map the reads to the reference genome. This mostly happens when there are gaps between sequence reads, where there is no overlapping. On the other side, LRS reads have completely overlapped sequences, which makes them easier in both genome alignment and *de novo* assembly. **(D)** *De novo* genome assembly: rare or unique mutations and SVs could be covered successfully by LRS reads, which results in better genome assembly for individual PGx genes. **(E)** Update the PGx references: LRS can add multiple aspects for content creation and/or updating in PGx reference organizations and databases as well as PharmVAR, PharmGKB, etc*.* **(F)** Drug hypersensitivity discovery: extremely rare and highly polymorphic PGx alleles (including HLA genes) are now covered and deciphered by the utilization of custom-made LRS-dependent HLA-typing approaches. PGx: pharmacogenomics; SNV: single-nucleotide variant; CNV: copy number variation; SV: structural variant.

LRS has facilitated advances in genomic and PGx studies by unveiling previously concealed pharmacovariants within core pharmacogenes and genes associated with pharmacogenetic pathways. A brief description of the potential impact of this technology on PGx testing is given below, showcasing opportunities that can be obtained through its application in both clinical and research settings. Also, Figure 2 provides a quick overview on such applications in PGx setting.

#### Complex genomic regions in pharmacogenes

Pharmacovariants in complex genomic regions are not always accurately detected by genotyping technologies that rely on either SRS or SNV arrays (van der Lee et al., 2020). LRS techniques have the ability to analyze complex PGx regions effectively (Wenger et al., 2019; Bowden et al., 2019; Midha et al., 2019). For example, LRS has successfully identified variants and haplotypes within complex pharmacogenes like *CYP2D6* and *HLA-A*, which contain repeats or segmental duplications. LRS technologies also have advantages in haplotype phasing of variants within complex loci (van der Lee et al., 2022), *de novo* genome assembly (Cai et al., 2020), and transcriptome assembly (Tilgner et al., 2014).

#### Haplotype characterization and resolving uncertain haplotype phasing

While linkage disequilibrium (LD) assessment is utilized for PGx diplotype phasing and can yield accurate haplotype phasing at the population level, its accuracy at the individual scale is limited (van der Lee et al., 2022). Other tools for rapid and accurate statistical phasing of large sequencing datasets with an error rate of <5% have been developed (Hofmeister et al., 2023). Statistical phasing does not require supplementary experiments, but it is contingent upon the availability of representative datasets encompassing population genetic information. Despite demonstrating notable precision at a local level, the inherent limitation of statistical phasing becomes apparent in the form of multiple errors (referred to as “switch errors”), particularly at recombination hotspots. This occurs because software that uses LD only considers the co-inheritance of alleles that are closely located along the chromosome (Xu and Dixon, 2020).

LRS does not require pedigree or computational approaches, which makes haplotyping more straightforward and higher throughput, thus improving the accuracy of genotype imputation (van der Lee et al., 2022) and enabling more accurate PGx phenotype prediction (van der Lee et al., 2020). LRS has the potential to generate fragments of DNA sequences long enough to decode an entire pharmacogene in a single read, such as the 6.6-kb *CYP2D6* gene (Zhou et al., 2022). LRS has been used to accurately identify the haplotype of complex genes such as *HLA* and *CYP2D6* (Ammar et al., 2015; Liau et al., 2019a). PacBio HiFi long-reads have also been used for direct phasing and haplotype calling of the *NUDT15* gene for more comprehensive diplotyping of star (*) alleles (Scott et al., 2022).

#### Resolving ambiguous genotype calls

LRS platforms also allow resolution of ambiguous and inconsistent calls that might be due to novel alleles and sub-allele calls (Gaedigk et al., 2022). Data obtained from 137 DNA samples recharacterized by employing the LRS technology and three genotype calling algorithms identified a previously unresolved *CYP2C19* diplotype. The result decoded two novel sub-alleles, CYP2C19*35.002 and CYP2C19*2.011, which were previously assigned as CYP2C19*1 (*15)/*2 (indeterminate metabolizer) but have been updated to CYP2C19*2/*35 (poor metabolizer). The findings from LRS also indicated that CYP2C9*10 and *12 alleles are not located in a *trans* configuration, as previously thought, but rather in a *cis* arrangement, giving rise to a new and unique haplotype (Gaedigk et al., 2022).

#### Updating the existing knowledge and adding novel PGx reference alleles

LRS has the potential to detect novel variants and generate accurate reference alleles in diverse populations. A reference genome was created by integrating *de novo* assemblies of three Japanese individuals using LRS technologies (Takayama et al., 2021). LRS alongside SRS was utilized to obtain the genome of an individual from Saudi Arabia for creating the first draft of the genome based on *de novo* genome assembly, which was then refined (Kulmanov et al., 2022).

The Egyptian Genome Reference (EgyptRef) project was initiated in 2020 using a combination of long- and short-read sequencing technologies, including 10x Genomics, Illumina, and Pacific Biosciences (PacBio). In this project, the data obtained from 109 individuals demonstrated 1,198 Egyptian population-specific variants, including 49 novel ones (Ateia et al., 2023). Another study on Arabs recently investigated the genomes of 43 individuals from diverse Arab ethnicities using an ultra-long-read sequencing kit (ULK), high fidelity (HiFi), PacBio, and ONT. The study aimed to construct the Arab pan-genome reference and resulted in the detection of millions of base pairs of novel sequences (Uddin et al., 2023). In previous studies, the investigation of the genomes of diverse populations using mostly array-based technologies has resulted in novel PGx discoveries. For instance, in a study on the Spanish population, a significant number of potentially damaging variants that were not previously associated with PGx were identified (Nunez-Torres et al., 2023). Another study discovered a new link between a genetic variation in *FPGS* and reduced the need for warfarin dosage in the African American population (Giri et al., 2014). Moreover, the association between a novel non-function variant, NQO1*2, and resistance to warfarin was discovered in a Puerto Rican case (Hernandez-Suarez et al., 2016). Moving forward, LRS is likely to enhance the feasibility of detecting novel variants and may be used to update PGx references, especially in diverse populations that are not well-studied with respect to PGx (Kaye et al., 2017; Venner et al., 2024).

LRS revealed novel alleles in *NUDT15*, which were confirmed by Sanger sequencing and assigned as *1.007 and *20 by the PharmVar Consortium (Scott et al., 2022). SMRT sequencing has also led to characterization of new alleles and tandem arrangement, configuring diplotypes like CYP2D6*36+*41, facilitating prediction of a metabolizer phenotype and rate of drug response (Qiao et al., 2016). Discovering novel haplotypes, alleles, and sub-alleles through LRS, once confirmed and clinically validated, can accelerate the updating of PGx references. In addition, such technologies may offer a more rapid time-to-result approach for a PGx or other targeted tests. A nanopore sequencing-based approach led to reduced turnaround time from approximately 7 days to 24 h (Oehler et al., 2023). This rapid integration of verified genetic information has the potential to enable healthcare professionals to make more informed decisions about drug prescriptions and dosages with more speed and accuracy.

#### *De novo* genome assembly in personalized medicine

The capability of LRS in providing *de novo* assemblies is especially valuable in PGx, where it allows for the identification of individual-specific genetic variation within pharmacogenes. This variation can be critical in understanding an individual’s response to drugs and predicting adverse reactions or treatment efficacy. An extensive example of population-based, LRS-aided *de novo* genome assembly was provided by the Seo group in the Korean population. A total of 18,210 SVs were identified by direct comparison of their assembly with the human reference, which led to the identification of thousands of previously unreported breakpoints. Asian-specific SVs were decoded, and high-quality haplotyping of clinically relevant alleles was provided alongside successful characterization of *HLA* and *CYP2D6* regions (Seo et al., 2016).

#### Long-read sequencing advantages in decoding PGx genes (proofs of principle)

LRS technologies are now widely used in locus characterization and for improving phenotype prediction in PGx investigations (Deserranno et al., 2023). Such studies successfully mitigated the ambiguity associated with challenging genotypes and missing heritability, resulting in conclusive findings. In this context, in addition to providing detailed examples of the application of LRS, focusing on the very important pharmacogenes *CYP2D6* and *HLA* as proof of concept, Table 3 provides a summary of recent studies focusing on utilization of LRS technologies in clinical PGx testing.

**TABLE 3:** Summary of studies with successful utilization of long-read sequencing technologies in clinical pharmacogenomics.

Study purpose	Samples’ phenotype	Genomic region	Platform	Average read depth/coverage	Sample size (n)	Summary of results	Ref
Establish a method for sequencing and haplotyping of the entire CYP2D6 gene using Nanopore sequencing	Adverse drug reaction	CYP2D6	GridION Nanopore	200–1,000X	78	Identified known and new alleles, including gene duplications	Liau et al. (2019a)
Explore *CYP2D6* gene variation in 202 Māori and Pacific individuals in New Zealand	Samples from two studies: Genetics of Gout, Diabetes, and Kidney Disease in Aotearoa New Zealand and Pasifika Heart study	CYP2D6	GridION Nanopore	>200X	202	Identified twelve new variants, including the relatively common CYP2D6*71 (8.9%)	Hitchman et al. (2022)
Improve the accuracy of *CYP2D6* genotyping	Samples from the CYPTAM study (Netherlands Trial Register 1,509)	CYP2D6	PacBio** RSII	>200X	24	Identified 61 unique variants and created a database to aid genomic analysis	Buermans et al. (2017)
Introduce a novel and reliable method for fully characterizing the *CYP2D6* gene, resolving complex genotypes, identifying novel alleles, and characterizing duplicated alleles in individuals with CNVs	Samples from Coriell Biorepository	CYP2D6	Single-molecule real-time (SMRT)	∼20,000X	25	The long-read *CYP2D6* SMRT sequencing method successfully identified, resolved, and discovered alleles	Qiao et al. (2016)
Enhancing drug safety and efficacy by enabling pre drug administration screening	Positive diagnosis of vivax malaria, negative for G6PD deficiency	CYP2D6	PacBio single-molecule real-time (SMRT)	500X	377	Provided detailed insights into the genetic variation of the *CYP2D6* gene in the Solomon Islands population and its potential impact on drug metabolism	Charnaud et al. (2022)
Improve *CYP2D6* genotyping with developing a genotyping assay called nCATS-CoLoRGen, combining nanopore Cas9-targeted sequencing and a new CoLoRGen pipeline	Two lymphoblast cell lines, HG01990 and GM19785, and NA12878 cell line	CYP2D6–CYP2D7 hybrid allele	MinION ONT***	7X-238X for different libraries	Three cell lines	Methods accurately identified star-alleles in three cell lines and revealed previously undetected variants	Rubben et al. (2022)
Analysis of pharmacogenetic variants related to clopidogrel and warfarin	Had undergone percutaneous coronary intervention (PCI)	CYP2C19 *B4GALT2 ABCB1 CYP2C9 GGCX CYP4F2 VKORC1*	MinION ONT	42–975X	81	Drug-specific or broad pharmacogenetic screening assays using small PCR amplicons are feasible on the MinION sequencing device	Liau et al. (2019b)
Emphasize the potential of long-read sequencing for understanding intricate pharmacogenes and maintaining precise variant calling	Data of the HG002 GIAB sample	100 Pharmacogenes	PacBio sequel	97.62X	Long-read sequencing results of GIAB sample HG002	73% of pharmacogenes were fully covered in one phased haploblock, including 9/15 highly complex genes. Most gene–drug interactions from DPWG and CPIC guidelines were fully resolved (62% and 63%, respectively)	van der Lee et al. (2022)
Develop a genotyping method for the complex *CYP2D* gene loci to improve drug therapy decision-making	Samples from Eunice Kennedy Shriver National Institute of Child Health and Human Development (NICHD) and Coriell Institute for Medical Research	The entire *CYP2D6*–*CYP2D7–CYP2D8* loci	PCR-free CRISPR-Cas9-based enrichment method for targeted long-read sequencing	>150X	—	Identified new *CYP2D6* variants and characterized *CYP2D7* and *CYP2D8* haplotypes	Turner et al. (2023)
Advance personalized medicine by improving pharmacogenomics, which tailors drug choices and dosages to individuals based on their genetic profiles with introducing a novel approach that uses continuous-scale assignments instead of categorical ones to predict enzymatic activity	A group of patients had breast cancer (took tamoxifen)	*CYP2D6*	PacBio Sequel	NP*	CYPTAM cohort: 608 subjects CYPTAM-BRUT cohort: 225 subjects Venlafaxine cohort: 78 subjects	Analyzed complete *CYP2D6* gene sequences and clinical data, a neural network model achieved better predictions (79% accuracy) compared to the conventional method (54%). This approach has potential to better predict individual drug responses	van der Lee et al. (2021)
Assess the use of long-read sequencing for pharmacogenomics research and clinical applications, focusing on the *CYP2C19* gene	Samples form PGx studies performed at the Leiden University Medical Center	Core PGx genes as well as some other genes with a focus on CYP2C19	PacBio Sequel	25.23X	48	Long-read sequencing identified 813 unique variants in *CYP2C19*, aiding in haplotype assignment	Graansma et al. (2023)
Create full-length personalized transcriptomes, detailing an individual’s genetic variants and transcript isoforms	NP	Full-length transcriptome	PacBio Illumina	NP	Three family members	The study revealed previously unknown splice sites, quantified new splicing isoforms, connected RNA molecules to their alleles, and assessed differential allelic expression and isoforms. Introduced the first comprehensive full-length personal transcriptome	Tilgner et al. (2014)
Assess the feasibility of using a rapid Nanopore-based method for HLA typing associated with drug hypersensitivity	Specimens obtained from GeT-RM (Coriell Institute for Medical Research) and Thai patients from a standard pharmacogenetics laboratory	HLA	ONT, ONT	NP	20	Method reduced hands-on time, from approximately 9 h–4 h The transposase-based sequencing method demonstrated the ability to report 3-field *HLA* alleles	Anukul et al. (2023b)
Develop an efficient method for genotyping *HLA-B* alleles, with a focus on New Zealand Maori and Pacific Island populations, to understand the prevalence of alleles associated with adverse drug reactions and disease susceptibility	Forty with no history of inflammatory disorders, and nine as references for the MinION analysis	*HLA-B* locus	ONT, ONT MinIONTM	5,807X	40	Used MinIONTM nanopore sequencer and SeqNext-HLA software to genotype *HLA-B* alleles in 49 DNA samples, achieving high-resolution results	Ton et al. (2018b)
Improve the characterization of highly polymorphic *HLA* genes	NP	HLA class I and class II	Single Molecule Real-time (SMRT) PacBio	NP	—	Introduced an end-to-end workflow using third-generation SMRT sequencing, for *HLA* Class I and Class II genes, enhancing the accuracy of *HLA* gene analysis	Ambardar and Gowda (2018)
Develop a long-read sequencing method to determine the phase of *NUDT15* gene variants for better allele diplotyping, enhancing *NUDT15* pharmacogenomic testing	Samples form 1000 Genomes Project v3, the Coriell Institute for Medical Research, and healthy Ashkenazi Jewish individuals	NUDT15	Single-molecule real-time (SMRT) PacBio	NP	—	Discovered new *NUDT15* alleles, potentially improving pharmacogenomic testing and phenotype prediction	Scott et al. (2022)
Establish accurate immunogenetic Reference panels for population-scale immunogenomics, focusing on the complex major histocompatibility complex (MHC), By integrating multiple sequencing technologies and specialized bioinformatics	MHC-homozygous cell lines	MHC Locus	MinION, GridION and PromethION ONT Illumina PacBio	∼9–85X for different platforms	Five MHC-homozygous cell lines	The study assembled six MHC haplotypes, including DR1 and DR4 structures, using advanced sequencing and bioinformatics	Houwaart et al. (2023)
Use long-read RNA sequencing to provide rapid HLA genotyping results and normalized HLA transcript expression	Patients undergoing evaluation for solid organ transplantation and healthy donors	HLA typing (HLA-A, -B, -C, -E, -F, G, -H -DRB1, -DRB3, -DRB4, -DRB5, -DQA1, -DQB1, -DPA1 -DPB1, MICA, and MICB loci.)	MinION ONT	NP	18	The assay showed a strong concordance rate of 99.7% and revealed differences in *HLA* transcript expression, including lower *HLA-C* expression compared to *HLA-A* and *-B* in class I loci	Cornaby et al. (2022)
Develop a long-read SMRT sequencing method to analyze the *SLC6A4* gene	Samples from the Coriell Institute for Medical Research	SLC6A4	PacBio single-molecule real-time (SMRT)	∼20000X	120	Successfully identified the S allele and phased the *SLC6A4* promoter haplotypes. LRS can accurately interrogate the low-complexity homologous *SLC6A4* promoter region	Botton et al. (2020)
Develop a cost-effective Nanopore sequencing pipeline for precise fine-mapping of genetic loci discovered through GWAS, focusing on identifying potential causal SNPs	Doxorubicin- exposed pediatric patients with cancer	*SLC28A3* locus	MinION ONT	—	6	Successfully demonstrated the utility of Nanopore sequencing for pinpointing a causal SNP associated with doxorubicin-induced cardiotoxicity. This approach offers accuracy and cost-effectiveness for post-GWAS fine-mapping, with a minimal cost of approximately $10 per 100 kb/sample	Magdy et al. (2020)
Evaluate the feasibility of incorporating real-time analysis of somatic mutations in 19 genes into the management of cancer patients	Histologically or cytologically diagnosed with advanced solid cancer, potential candidates for phase I/II clinical trials, at least one biopsiable lesion, laboratory Parameters safe for tumor biopsy	The panel consists of 24 multiplex assays that detect 238 mutations in 19 oncogenes	PacBio RS	600X	50	Analyzed samples from 50 patients, identifying 19 actionable mutations in 16 patients	Tran et al. (2013)

#### Long-read technologies and CYP2D6

With more than 170 frequently updating listed star (*) alleles, the cytochrome P450 2D6 (*CYP2D6)* gene is considered highly polymorphic. Recombination events generate gene deletions, duplications, and conversions, and the *CYP2D6* pseudogene *CYP2D7* also impacts the *CYP2D6* structure (Kramer et al., 2009a; Kramer et al., 2009b). Hence, there is a high level of hybrid alleles and CNVs with clinical consequences in diverse populations. Furthermore, when a higher copy number is found, identification of duplicated alleles is required for reliable prediction of the *CYP2D6* metabolizer status. In the SUPER-Finland study, the importance of identification of *CYP2D6* copy number is highlighted in a large population with psychiatric disorders. A higher frequency of *CYP2D6* ultrarapid metabolizers and a substantial enrichment of specific variants were observed within the Finnish population (Häkkinen et al., 2022). The potential benefit of long-read *CYP2D6* sequencing is also indicated by the ability to reduce multiple alignments in single-end reads larger than 3 kb (Yang et al., 2017).

Hitchman et al. utilized nanopore sequencing for *CYP2D6* alterations in 202 New Zealanders and reported 12 novel variants in this population (Hitchman et al., 2022). Buermans et al. introduced PacBio’s RSII with a two-step barcoding approach as a high-quality and cost-efficient assay for full-length *CYP2D6* sequencing and haplotyping. They reported 61 unique alterations in *CYP2D6* (Buermans et al., 2017). In a Solomon Islands population, the “PLASTER” (Phased Long Allele Sequence Typing with Error Removal) pipeline identified one of eight * alleles in 95.3% of patients and seven new * alleles in 4.7% patients, using long-read SMRT sequencing (Charnaud et al., 2022). Nanopore sequencing has also been used for high throughput and accurate haplotyping and identifying novel alterations and CNVs in challenging pharmacogenes like *CYP2D6*. A whole gene deletion (*5) along with 2484G>T (*33) variants were observed in one patient and confirmed on Sanger sequencing (*5/*33), resulting in an intermediate metabolizer phenotype. Furthermore, five novel rare variants have been found, including *17.003 (*17 allele with a 653C>T intronic variant), *41.004 (two variants of 1378C>G and 5814C>T in intron 2 and exon 3, respectively), *4.026 (contained 1404C>T in intron 2), and *1.025 (−365G>A) (Liau et al., 2019a). Rubben et al. introduced a new PCR-free nanopore Cas9-targeted sequencing (nCATS) for genotyping of allele-specific sequences of complex regions and a new extensive LRS (CoLoRGen) pipeline. These assays are efficient and accurate methods for determination of the complete *CYP2D6*–*CYP2D7* sequence (Rubben et al., 2022). Ammar et al. demonstrated that MinION nanopore sequencer-based LRS technology can identify both alterations and haplotypes of key pharmacogenes including *HLA-A*, *HLA-B,* and *CYP2D6*. Because of the non-functional CYP2D6 enzyme associated with *3 and *4 alleles, decreased metabolism of codeine and olanzapine has been observed (Ammar et al., 2015). Several sub-alleles have been found in *CYP2D6*, which may cause misalignments in short-read NGS data. For example, rs61736524 (G>A) in exon 4 of *CYP2D8* adjusts rs748851484 (G>A) in exon 4 of *CYP2D6*. To handle *CYP2D6* and the related pseudogenes, single-molecule LRS has been used for the entire *CYP2D6*–*CYP2D7*–*CYP2D8* locus, indicating that it could overcome the limitation and challenges of other genotyping assays like short-read NGS and SNP-arrays (10). Ten novel *CYP2D6* haplotypes, including *128 to *137 alleles, were identified by a targeted long-read panel (PKSeq) and confirmed *in vitro* metabolic studies in a Japanese population and suggested to be nonfunctional alleles. In addition, the *135/*136 diplotype was described as a reduced function phenotype. The alleles *129, *134, *135, *136, and *137 all have the potential to create a reduced functional *CYP2D6* phenotype through the decreased CYP2D6 activities (Fukunaga et al., 2021).

#### Long-read technologies and HLA genes

Variation in human leukocyte antigen (HLA) genes is considered a strong marker for multiple drug hypersensitivity reactions (Karnes et al., 2019). HLA typing at a high resolution can be clinically useful in predicting severe cutaneous adverse drug reactions to certain drugs (Fan et al., 2017). Hypersensitivity to abacavir and carbamazepine is associated with the carriage of *HLA*-B*57:01 (Illing et al., 2018) and *HLA*-B*15:02, respectively (Leckband et al., 2013). *HLA*-B typing can aid healthcare providers in selecting suitable drugs and mitigating the risk of adverse drug effects (Matern et al., 2020).

HLA typing methods have shifted from serologic testing to DNA sequencing. Because of extreme polymorphic zones and uncommon population-specific variation, it is challenging to choose an effective sequencing strategy. Recently, LRS was validated for HLA by Benedict et al. They reported a method for sequencing and typing of full-length HLA class-I molecules using the Oxford Nanopore MinION and the implementation of this method in a routine diagnostic setting (Matern et al., 2020). Ultrarapid and high-resolution HLA class-I typing using transposase-based nanopore sequencing was also applied in PGx testing by other groups, its potential for conducting testing that is independent of race and population was demonstrated, while significantly reducing both time and costs (Anukul et al., 2023a). However, the accuracy of typing results could be impacted by an imbalance in the PCR amplification of distinct haplotypes (Anukul et al., 2023a). Ton et al. outlined a method for *HLA-B* typing that combines data analysis with the SeqNext-HLA software package with NGS on the MinION™ nanopore sequencer. Alleles within this specific gene are described as crucial risk elements for hypersensitivity reactions triggered by drug intake. Researchers illustrated effective haplotyping facilitated by the nanopore sequencer’s long-read capacity and single-molecule reads. The *HLA-B* diversity in Pacific Islander and Maori population is not well-characterized, so this approach was utilized in both of these populations as well as reference samples (Ton et al., 2018a).

The Pac-Bio SMRT sequencing technology yields fully phased, clear, allele-level data for full-Length *HLA* typing (Ambardar and Gowda, 2018). Given the time limits for allocating deceased organ donors, a test that delivers both rapid allele-specific transcript quantitation and high-resolution HLA typing with LRS technologies is ideal. Overall, *HLA* typing with the accuracy of 99.68% is demonstrated by this method (Cornaby et al., 2022).

#### Long-read technologies and other pharmacogenes

Other PGx regions have been phased into haploblocks, mediated by long-read PacBio sequencing. The total *CYP2D6* locus in one long read was covered using PacBio, and variants in other genes like rs776746 (6981A>G) of *CYP3A5* and 1173 TT genotype (homozygous for 6484C>T) in *VKORC1* lead to poor metabolizer and decreased activity phenotypes, respectively, and can be identified with LRS technologies (van der Lee et al., 2022). Other studies demonstrated a higher rate of on-target mapped nucleotides in pharmacogenes, which was seen in nanopore sequencing compared to Illumina, resulting in the longer length of sequencing (e.g., in *CYP2C19* and *CYP2D6*) (Tilleman et al., 2022). Graansma et al. identified 813 unique variants in 37 samples and showed that the package of LRS and PGx-dedicated bioinformatics tools (*Aldy, PharmCAT,* and *PharmKU*) can recognize *CYP2C19* variation and new haplotypes (Graansma et al., 2023). LRS affords high coverage of regions within these genes and addresses the high level of heterozygosity in individuals (Liau et al., 2019b).

*NUDT15* encodes a Nudix hydrolase enzyme that is a negative regulator of thiopurine activation and toxicity. Mutations in this gene may lead to poor metabolism of thiopurines. In contrast to short-read genome sequencing, which was unable to phase star (*) allele-defining *NUDT15* variants, long-read HiFi sequencing phased all variants across the *NUDT15* amplicons, including the *2/*9 diplotype. This diplotype is interpreted as a possible poor metabolizer, which may pose the patient to high risk for toxicity with normal doses of thiopurines. As a result, this method was considered an innovative platform for allele discovery and new full gene haplotyping (Scott et al., 2022). Other examples include the serotonin (5-HT) transporter (5-HTT), produced by the *SLC6A4* gene, which regulates 5-HT reuptake and is known as the major target of selective serotonin reuptake inhibitor (SSRI) antidepressants. The promoter of *SLC6A4* consists of a variable number of homologous 20–24bp repeats (5-HTTLPR) and has rare population-specific alterations. A long-read SMRT sequencing method can provide an efficient technology for the investigation of the *SLC6A4* promoter region that could not be determined using high-depth short-read capture-based sequencing (∼330X) (Botton et al., 2020). Solute carrier family 28 member 3, *SLC28A3*, impacts neurotransmission and nucleoside drug metabolism and transport. Recently, nanopore long-range PCR-based target enrichment for *SLC28A3* amplicons through fine-mapping of a genome-wide association study (GWAS) revealed an association between an SNP (rs7853758G>A, L461L) in *SLC28A3* and lower risk of cardiotoxicity in patients treated with doxorubicin. This investigation also provides a proof of principle for the application of LRS in pinpointing potential causal SNPs that could be used to optimize pharmacotherapy (Magdy et al., 2020).

### Challenges and limitations in utilization of long-read sequencing for clinical practice and pharmacogenomic testing

Current LRS technologies have several limitations in both infrastructure and data production and/or management. At present, LRS instruments and reagents are more expensive, and it is not a cost-effective approach for many test centers (Amarasinghe et al., 2020b). Library preparation requires fresh material or intact cells, and there is a need for improvements in DNA isolation protocols and handling of ultra-long high-molecular weight DNA (Mantere et al., 2019). Obtaining high-quality DNA samples can be a challenging aspect of LRS, especially when dealing with specific sample sources such as degraded or low-yield samples. In addition, formation of PCR chimeras during PCR amplification and biases in reference alignment are challenges that must be taken into account when trying to phase variants with amplicon-based LRS technologies (Laver et al., 2016). An example of this issue is demonstrated by Ammar et al., who determined the *HLA-A* and *HLA-B* haplotypes in a manner similar to the *CYP2D6* haplotypes, utilizing predefined markers from the HapMap project. Taking into account the errors in MinION sequencing and allowing for a single mismatch in the haplotype, the investigated diplotype for those genes revealed PCR bias during amplification, which likely influenced the relative proportion of haplotypes. Owing to the high error rates of nanopore reads, the identified *HLA* alleles did not match those found using HapMap data (Ammar et al., 2015).

Recent advancements in some platforms like PacBio HiFi produce 99.9% accuracy in long DNA sequencing reads and significantly decreased error rates (Hotaling et al., 2023). However, in general, these technologies have a higher rate of sequencing errors, ranging from 5% to 20%, compared to short-read next-generation sequencing (SR-NGS) outcomes, which typically have an error rate of less than 1% (Wenger et al., 2019; Goodwin et al., 2016). LRS errors are generally randomly distributed. These issues have the potential to affect the quality of assembled sequences and the accuracy of variant calling (Amarasinghe et al., 2020a).

Analyzing LRS data presents a significant computational challenge. Specialized bioinformatics tools and expertise are frequently essential for tasks such as error correction, alignment, and *de novo* assembly. These processes can be resource-intensive and demand substantial computational power and expertise (Ozsolak, 2012). Managing and archiving these large volumes of data can pose logistical challenges, especially for long-term projects that require data preservation and accessibility over extended periods (De Coster et al., 2021). Reference genomes that currently exist are primarily constructed using SRS data. When long reads are aligned to these reference genomes, it is important to be aware that biases can emerge, potentially resulting in misalignments and errors in variant calling (Sedlazeck et al., 2018b).

Achieving sufficient coverage depth and accurate *de novo* assembly could be considered an emerging challenge for LRS utilization in PGx studies. Although LRS shows unprecedented ability for uncovering genomic base modifications as well as DNA methylation, accurately pinpointing the precise location and type of these modifications presents another challenge. Specialized analysis methods are typically necessary to achieve accurate detection and characterization of base modifications through DNA samples (Eid et al., 2009; Johannesen et al., 2023). Finally, it is crucial to evaluate the accuracy and reliability of the results from long-read sequencing technologies, especially in clinical practice. Common orthogonal methods to validate findings from long-read sequencing include Sanger sequencing and quantitative RT-PCR, which are typically conducted to validate results in the proband and their family. These methods are also utilized to validate the results and refine the exact position of breakpoint junctions. Meanwhile, an automated quality control tool named *LongQC* is designed specifically for genomic datasets produced by third-generation sequencing technologies, including ONT and SMRT sequencing from PacBio, and it supports all major LRS platforms. Moreover, structural variant visualization tools, such as Ribbon, facilitate easy manual validation of SVs, allowing researchers to verify findings without relying solely on SV calling tools (https://v2.genomeribbon.com/) (see the Supplementary Table S2 for more details).

## Future trends and conclusion

Despite the existing challenges and limitations, integration of LRS technologies into PGx testing is continually evolving, resulting in new possibilities for individualized treatment. Access to such technologies is increasing as an outsource point for many clinical and academic sections through the service providers, offering PacBio and ONT sequencing facilities and data analysis. Supplementary Table S3 introduces many of reliable and well-known centers, offering collaborations in this field. Future LRS-based PGx studies are likely to benefit from personal genome assembly, rapid decoding of novel and rare/underrepresented variants within core pharmacogenes, comparing ancestrally diverse samples, patient-specific primer design and optimization for challenging pharmacogenes, detecting and phasing *de novo* CNV and SV haplotypes, and resolution of previously ambiguous bases (Pirmohamed, 2023; Giardina and Zampatti, 2022). Fourth-generation (4G) sequencing machines with the ability for real-time sequencing of nucleotides in the fixed cells and tissues were also introduced through integration of nanopore sequencing into the single-molecule sequencing method. Advantages include the broader look into the arrangement of DNA reads across the sample, offering valuable insights into tissue heterogeneity using known markers. Moreover, the technology may lead to an ultrafast scan of the whole genome in 15 min (Pervez et al., 2022).

Such advancements are expected to be introduced into PGx testing as well. Current applications of LRS in stratified medicine utilizes drug-/treatment-related PGx panel for long-read technologies in order to find true gene–drug interactions in individuals. As the technology moves forward, more LRS-WGS will likely be performed as the predominant method for PGx profiling. The outcome may help in faster PGx implementation in clinical decision and drug stratification. Pre-emptive genotyping strategies may facilitate improvement of drug safety and efficacy, while reducing hospitalization and improving cost effectiveness. Other factors as well as sex, age, co-morbidity, drug–drug interactions, and organ functionality contribute significantly in overall drug response variability within individuals, yet genetic variation may constitute a crucial influence on those differences (Caspar et al., 2021). Comprehensive PGx marker screening and digitalization of the result for convenient and widespread access by healthcare professionals may facilitate utilization of the data for prescription modifications. To achieve this, the complexities in genome structure and function will need to be addressed by more advanced genotyping platforms. LRS technologies are promising tools to reach these goals through advancement in introducing highly accurate platforms (e.g., HIFI technologies) and compound bioinformatic algorithms to bring more precise genotype–phenotype correlations, aiding in PGx clinical evidence generation. This precision is particularly vital in modern pharmacology, where LRS can help guide therapeutic decisions, ensuring that patients receive medications tailored to their genetic makeup, maximizing both safety and efficacy.

## Mini glossary

**Complex genomic regions:** genomic regions with intricate structural variations, making them challenging to analyze due to tandem repeats, duplications, deletions, and rearrangements. In addition, they often involve multiple genes/pseudogenes and regulatory elements, leading to complex phenotypic traits.

**Circular consensus sequencing:** a method that generates highly accurate DNA sequences by repeatedly reading circularized DNA fragments, reducing sequencing errors. Sequencing of the same DNA fragment in a circular manner, ensuring consensus on the base calls.

**Diplotype:** the combination of two distinct haplotypes for specific genes or genomic regions in an individual’s genome. The specific pair of alleles present at two linked loci on homologous chromosomes in a diploid organism, representing a combination of two distinct haplotypes.

**Fine mapping:** a detailed genetic mapping approach to pinpoint the precise location of a gene or variant associated with a specific trait or disease. It is a refined mapping approach that aims to identify the exact genetic variants contributing to a specific phenotype by narrowing down the location within a previously identified genetic region.

**Fourth-generation of sequencing:** cutting-edge sequencing technologies that surpass previous generations, often characterized by higher throughput, lower costs, and improved accuracy. The advanced sequencing methods that utilize innovative approaches, such as nanopore sequencing or single-molecule sequencing, characterized by improved speed, accuracy, and scalability.

**Gene activity score:** a numerical measure, often derived from high-throughput sequencing data, quantifying the level of gene expression or activity in a specific biological context, mostly used in genomic research.

**Gene conversion:** the process where the genetic material is transferred from one DNA molecule to another, leading to the exchange of genetic information. A recombination process where one allele replaces a homologous allele, leading to the transfer of genetic material between non-identical genes.

**Genetic mapping:** the process of determining the relative positions of genes or other genetic markers on a chromosome and establishing the distances between them. Genetic mapping can be done through various techniques, such as linkage analysis in families, association studies in populations, or physical mapping using DNA sequencing methods.

**Genome-wide association studies:** large-scale investigations exploring genetic variations across the entire genome to identify statistically significant associations between specific genetic markers and complex traits or diseases.

**Genomic VCF (GVCF):** genomic variant call format, a standard file format storing genetic variations (SNPs, indels, *etc.*) with associated metadata, commonly used in genomics research.

**Haplotype:** a set of genetic variations, or alleles, on a chromosome that are inherited together from a single parent due to low recombination frequencies.

**Haploblock:** a genomic region where genetic variations are inherited together due to low recombination rates, often used in association studies to identify linked variants. A contiguous genomic region with limited historical recombination, containing a set of closely linked genetic variants.

**Hybrid gene:** a gene formed by the fusion of sequences from two different genes, resulting from chromosomal rearrangements or translocations, leading to a novel genetic sequence with potential functional implications.

**Long-read sequencing:** sequencing technologies capable of reading longer DNA fragments, enabling the assembly of complex genomes, allowing for the analysis of complex genomic regions and the detection of structural variations.

**Multiallelic CNV:** copy number variants (CNVs) involving multiple alleles, indicating variations in the number of copies of a specific genomic segment among individuals.

**Seed-and-chain framework:** a computational algorithm used in sequence alignment, where short, exact matches (seeds) between sequences are identified and extended into longer alignments (chains) to detect similarities and differences between biological sequences, commonly used in genomics for sequence comparison and analysis.

**Star allele:** a specific variant of a gene associated with pharmacogenetic traits, often denoted with an asterisk (*), indicating distinct allelic forms that influence an individual’s response to drugs or other substances.

**Zero-mode waveguides:** Zero-mode waveguides (ZMWs) are nanoscale observation chambers used in single-molecule real-time (SMRT) sequencing technologies. These tiny wells confine the sequencing reaction to a volume so small that only a single DNA polymerase molecule and its associated template DNA strand can fit. This isolation enables real-time observation of the DNA synthesis process at the single-molecule level, contributing to the accuracy and efficiency of the sequencing method.

## Footnotes

1 Short interspersed elements

2 Long interspersed elements

3 Variable number tandem repeats

## Abbreviations

4G sequencing, fourth-generation of sequencing; API, application programming interface; BAM, binary alignment mapped; CCS, circular consensus sequencing; CLI, command line interface; CNV, copy number variation; CRAM, compressed reference-oriented alignment map; GDF, genotype data file; GIAB, Genome in a Bottle; GVCF, genomic VCF; LD, linkage disequilibrium; LRS, long-read sequencing; NGS, next-generation sequencing; SMRT, single-molecule real-time; SNP, single-nucleotide polymorphism; SNVs, single-nucleotide variants; SRS, short-read sequencing; SVs, structural variants; VCF, variant call format.
