# Supplementary material for: Leveraging long-read sequencing technologies for pharmacogenomic testing: applications, analytical strategies, challenges, and future perspectives
Source: Front Genet. 2025 Apr 30;16:1435416. doi: 10.3389/fgene.2025.1435416 (PMC12075302; doi:10.3389/fgene.2025.1435416)
Supplement: Supplementary file 1 [file Table1.docx]

**Supplementary Table1:** Commonly used bioinformatics tools and algorithms for converting raw LRS data into PGx-annotated output.

| Tool/Algorithm | Application | Repository/Documentation | Reference |
| --- | --- | --- | --- |
| *PacBio raw data processing:* |  |  |  |
| bash5tools.py | Converting h5 or POD5 to Fastq files | http://132.248.34.22:8080/smrtanalysis/doc/bioinformatics-tools/pbh5tools/doc/index.html | N/A |
| Dextractor | Converting h5 or POD5 to Fastq files (with the ability to set a cutoff score when -q parameter added (e.g., quality cutoff of 0.75-0.8)) | https://github.com/thegenemyers/DEXTRACTOR | N/A |
| Canu | Used for multiple manipulation of PacBio data from basecalling to read alignment | https://canu.readthedocs.io/en/latest/  https://github.com/marbl/canu | [136] |
| Cmph5tools.py | Used for multiple manipulation of PacBio data from basecalling to read alignment | http://132.248.34.22:8080/smrtanalysis/doc/bioinformatics-tools/pbh5tools/doc/index.html | N/A |
| SequelTools | Consists of three sub-tools for running quality control (QC), including read subsampling and filtering, read arrangements, and data normalization | https://github.com/ISUgenomics/SequelTools | [137] |
| *PacBio read aligners:* |  |  |  |
| Minimap2 | Used to align LRS reads against reference genomes. Integrated into Dorado tool as well. | https://github.com/lh3/minimap2 | [28] |
| Pbalign | Platform-specific algorithms for aligning LRS reads against reference genomes. It is a python wrapper for producing PBBAM valid alignments. | http://132.248.34.22:8080/smrtanalysis/doc/bioinformatics-tools/pbalign/doc/index.html  https://github.com/PacificBiosciences/pbalign | N/A |
| Pbmm2 | A SMRT C++ wrapper for Minimap2, which outperforms other PacBio-specialized aligners in terms of sequence identity, the count of mapped bases, and runtime efficiency | https://github.com/PacificBiosciences/pbmm2 | [16] |
| BLASR | PacBio-specialized aligner tool | https://github.com/mchaisso/blasr/tree/master | N/A |
| *PacBio mark duplication tool:* |  |  |  |
| Pbmarkdup | Sequence duplication removal tool with PacBio BAM (.ccs.bam) or PacBio dataset (.consensusreadset.bam) as input files. | https://github.com/PacificBiosciences/pbmarkdup | N/A |
| *ONT raw data processing:* |  |  |  |
| *Dorado* | Used for multiple manipulation of ONT data from basecalling to read alignment. Dorado is the default basecaller tool for converting POD5 to fastq file with the Enhanced data processing efficiency and accuracy. It is a data processing toolkit that includes alignment, modified base detection, and Barcode demultiplexing as well as normal basecalling. | https://github.com/nanoporetech/dorado | N/A |
| *MinKNOW* | Controls all Oxford Nanopore sequencing devices, performing several core tasks, including data acquisition, real-time analysis, basecalling, and data streaming. | <https://nanoporetech.com/document/experiment-companion-minknow>  https://github.com/nanoporetech/minknow_api | N/A |
| Guppy | ONT basecaller tool. It is used for converting Fast5 format to Fastq. The tool was a neural network basecaller, which also could filter and remove low quality reads, clip ONT adapters, and estimate the probability of methylation signature for each base. | https://denbi-nanopore-training-course.readthedocs.io/en/latest/basecalling/basecalling_1.html | N/A |
| Scrappie | ONT basecaller tool. | https://github.com/nanoporetech/scrappie#scrappie-basecaller | N/A |
| Abalcore | ONT basecaller that identifies DNA sequences directly from raw data, rather than utilising an intermediary stage called ‘event detection’. This upgrade enhances accuracy of the single-read sequence data, contributing to high consensus accuracy for nanopore sequence data. | https://github.com/dvera/albacore | N/A |
| IONiser | MinION-specific tool for QC steps. | https://github.com/grimbough/IONiseR | N/A |
| Minion_qc | MinION and PromethION dedicated tool for QC step | https://github.com/roblanf/minion_qc | [138] |
| PycoQC | Performing QC step for all types of ONT data. | https://github.com/a-slide/pycoQC | [139] |
| NanoPlot | Performing QC step for all types of ONT data. | https://github.com/wdecoster/NanoPlot | N/A |
| NanoQC | Performing QC step for all types of ONT data. | https://github.com/duceppemo/nanoQC  https://github.com/wdecoster/nanoQC | N/A |
| NanoFlit | A tool for ONT adapter trimming. | https://github.com/wdecoster/nanofilt | N/A |
| NanoPolish | A tool for detecting base modifications (e.g., methylated bases). | https://github.com/jts/nanopolish | [140] |
| *ONT read aligners:* |  |  |  |
| BBMAP | General genome aligner, applicable to both short-read and long-reads. | https://jgi.doe.gov/data-and-tools/software-tools/bbtools/bb-tools-user-guide/bbmap-guide/  https://github.com/BioInfoTools/BBMap | [141] |
| Minimap2 | General genome aligner, applicable to both short-read and long-reads. | https://github.com/lh3/minimap2 | [28] |
| NGMLR | LRS-specific genome aligner tool. It employs a k-mer search method that is sensitive to structural variations (SV) to identify approximate mapping positions for a read. It also utilizes a banded Smith-Waterman alignment algorithm to calculate the ultimate alignment. | https://github.com/philres/ngmlr | [33] |
| *LRS phasing tools:* |  |  |  |
| WhatsHap | A tool for direct haplotype phasing of short-read and long-read WGS genotyping results. | https://whatshap.readthedocs.io/en/latest/ | [34] |
| HapCUT2 | HapCUT2 uses sorted BAM files from Minimap2 for direct haplotype phasing of long-read sequencing data. | https://github.com/vibansal/HapCUT2 | [142] |
| LRphase | Recently developed tool which utilizes haplotype-resolved heterozygous variants, derived from various genomes including maternal and paternal genomes, in two different modes of scoring and matching to the observed genotype. | https://pypi.org/project/LRphase/ | [143] |
| PEPPER- Margin | Is a phasing and haplotyping method that takes the SNPs reported by PEPPER-SNP and generates a haplotagged alignment file using a hidden Markov Model (HMM). Margin takes the output of DeepVariant and the alignment file to generate a phased VCF file using the same HMM. | https://github.com/kishwarshafin/pepper | [144] |
| *LRS variant calling:* |  |  |  |
| Medaka (just for ONT) | Assembly error correction tools for ONT, which significantly reduce errors and prepare consensus sequences for LRS variant calling.  Medaka also can create consensus sequences and variant calls out of nanopore sequencing data directly. | https://github.com/nanoporetech/medaka | N/A |
| DeepVariant | General LRS variant callers that also evaluated for PGx variant calling and consistently demonstrated superior performance and robustness. | https://github.com/google/deepvariant | [145] |
| Clair3 | General LRS variant callers that also evaluated for PGx variant calling and consistently demonstrated superior performance and robustness. | https://github.com/HKU-BAL/Clair3 | [146] |
| Sniffles2 | A tool For SV detection, which utilizes an innovative scoring system which considers factors such as position, size, type, and coverage of candidate SVs to exclude false calls. This approach effectively mitigates the high indel error rates in LRS results, ensuring precise detection of SVs in both germline and somatic variation in population-level analyses for PacBio and ONT read data. | https://github.com/fritzsedlazeck/Sniffles | [33] |
| SVIM | Another common variant caller for LRS data is SVIM (pronounced swim) with the ability to mark and classify six types of SVs including: insertions, deletions, tandem duplications, interspersed duplications, inversions, and translocations. | https://github.com/eldariont/svim  https://github.com/eldariont/svim-asm | [147] |
| SMRTlink PbSV (just for PacBio) | PacBio-specific variant caller, which accepts pre-processed data named as SvSIG. | https://github.com/WenchaoLin/SMRT-Link | N/A |
| EViNCe (just for ONT) | ONT-dedicated variant caller tool. | https://github.com/davidebolo1993/EViNCe | N/A |
| human variation workflow  (just for ONT)  (epi2me-labs/wf-human-variation) | Current standard ONT variant calling pipeline on NextFlow platform, which can perform basecalling of Fast5 and calling all types of variants in ONT reads, including SNVs, SVs, methylation signatures, CNVs, and short tandem repeats (STRs) simultaneously. | https://github.com/epi2me-labs/wf-human-variation  https://labs.epi2me.io/workflows/wf-human-snp/ | N/A |
| NanoCaller (just for ONT) | Recently-introduced tool, uses deep learning and neural networks to call novel variants within complex genomic regions. | https://github.com/WGLab/NanoCaller | [41] |
| Longshot (just for ONT) | An older variant caller tool which does the accurate variant calling in diploid genomes from single-molecule long read sequencing. | https://github.com/pjedge/longshot | [148] |
| *LRS PGx variant calling:* |  |  |  |
| Aldy4 | Recent versions accept different types of sequencing data, including LRS, using new phasing strategies and improved star-allele calling models. | https://github.com/0xTCG/aldy | [44] |
| Cyrius | A computational algorithm in the list with built in compatibility for decoding LRS variants. Superior, accurate genotyping of CYP2D6 compared to other existing methods as well as Aldy and Stargazer. All types of variants and haplotype calling in addition to the structural and homology analysis will be covered for both GRCh38 and 37 genome builds. | https://github.com/Illumina/Cyrius | [45] |
| PyPgx | A python package works with both short-read and long-read sequences. alongside SNVs from SNP array data. Through a machine learning-based approach, PyPGx will predict star-allele calling. | https://github.com/sbslee/pypgx | [47] |
| Stargazer | Calls the star alleles from the NGS data by detecting SNVs, InDels, and structural variants. Stargazer detects  variations with structural changes including gene duplications, deletions, and conversions by calculating the paralog-specific copy numbers from read depth. Recently the tool has been replaced by PyPGx. | https://stargazer.gs.washington.edu/stargazerweb/ | [46] |
| PharmCAT | Comprehensive star-allele calling tool which can be used for long-read sequencing data as well despite of it’s limitations. See the main text for more details. | https://pharmcat.org/  https://github.com/PharmGKB/PharmCAT | [149] |
