# Supplementary material for: Leveraging long-read sequencing technologies for pharmacogenomic testing: applications, analytical strategies, challenges, and future perspectives
Source: Front Genet. 2025 Apr 30;16:1435416. doi: 10.3389/fgene.2025.1435416 (PMC12075302; doi:10.3389/fgene.2025.1435416)
Supplement: Supplementary file 2 [file Table2.docx]

**Supplementary Table2:** Tools and tests used to validate results of long read sequencing.

| Test Type | Required Tools | Assessed Variant Type | Reference |
| --- | --- | --- | --- |
|  |  |  |  |
| Sample QC*  Platform QC | LongQC  LongQC | Fastq, Fasta and subread BAM from PacBio  Fundamental stats for a run such as length or productivity in PacBio and some plots for productivity check in ONT. | https://github.com/yfukasawa/LongQC  (150) |
| Visualization | Ribbon | SVs* | (151) |
| Sanger Sequencing | - | Small-scale deletions and novel exons plus position of breakpoint junctions | (151, 152) |
| Quantitative RT-PCR | - | Small-scale deletions and novel exons plus position of breakpoint junctions | (151, 152) |

**QC: Quality control*

**SV: Structural variant*
