# Supplementary material for: Leveraging long-read sequencing technologies for pharmacogenomic testing: applications, analytical strategies, challenges, and future perspectives
Source: Front Genet. 2025 Apr 30;16:1435416. doi: 10.3389/fgene.2025.1435416 (PMC12075302; doi:10.3389/fgene.2025.1435416)
Supplement: Supplementary file 3 [file Table3.docx]

**Supplementary Table3:** Several certified service providers offer PacBio and ONT sequencing facilities. These are reliable and quality measured centers, which offering the utilization of LRS* platforms in both clinical and research studies.

| Technology | Location | Platform | Offering Center |
| --- | --- | --- | --- |
| *PacBio* | United States, Genomic Services Facility in North Carolina | Sequel IIe  Revio | 1. Eremid Genomic Services, PacBio Sequencing Services, 2025  2. University of Washington, PacBio Sequencing Services, 2025  3. Psomagen, Psomagen Joins PacBio Certified Service Providers for Revio, 2025  4. Leiden University Medical Center (LUMC), Leiden Genome Technology Center (LGTC), 2025  5. QB3 Berkeley, Pacific Biosciences Sequel Library Generation and Sequencing Services, 2025  6. Stanford Genome Technology Center, PacBio Sequencing Services, 2025  7. Center for Advanced Technology, UCSF, Services, 2025  8. Wellcome Sanger Institute, genomic services, 2025  9. Broad Institute of MIT and Harvard, Pacific Biosciences Iso-Seq, 2025 |
|  | United States, Research University in Seattle, Washington | Revio |  |
|  | United States, Biotechnology Center in Maryland | Revio |  |
|  | Netherlands, University in Leiden | Sequel II  Revio |  |
|  | United States, University of California, Berkeley, Stanford and San Francisco | Sequel II  Sequel IIe  Revio |  |
|  | United Kingdom, British genomics and genetics research institute in Hinxton | Sequel II  Sequel IIe  Revio |  |
|  | United States, biomedical and genomic research center in Cambridge, Massachusetts | Sequel II  Revio |  |
| *Oxford Nanopore Technology* | United States, University of California, Santa Cruz | PromethION  MinION | 1. UC Santa Cruz Genomics Institute, Sequencing Technology center, 2025  2. University of Wisconsin-Madison Biotechnology Center, Oxford Nanopore Technologies, 2025  3. Eurofins Genomics, Oxford Nanopore Sequencing, 2025  4. Wellcome Sanger Institute,  genomic services, 2025  5. University of Oxford, Centre for Human Genetics, 2025 |
|  | United States, University-Based Genomics Center in Madison, Wisconsin | PromethION |  |
|  | Europe, Biotechnology Research Facility in Germany, UK, and France | PromethION  MinION  GridION |  |
|  | United Kingdom, British genomics and genetics research institute in Hinxton | PromethION  MinION |  |
|  | United Kingdom, university in Oxford | PromethION  MinION  GridION |  |

**LRS: Long-read sequencing*
